# Veränderungen der Kontextfaktoren und deren Auswirkungen auf die Arzt-Patient-Beziehung

**DOI:** 10.1007/s00739-021-00774-5

**Published:** 2022-01-05

**Authors:** D. Steinmair, A. Ronge-Toloraya, H. Löffler-Stastka

**Affiliations:** 1 Klinik für Psychoanalyse und Psychotherapie, Medizinische Universität Wien, Währinger Gürtel 18–20, 1090 Wien, Österreich; 2 Karl Landsteiner Privatuniversität für Gesundheitswissenschaften, Dr. Karl-Dorrek-Straße 30, 3500 Krems, Österreich; 3 Klinische Abteilung für Augenheilkunde, Universitätsklinikum St. Pölten, Dunant-Platz 1, 3100 St. Pölten, Österreich; 4 Unit für Postgraduelle Aus- und Weiterbildung, Medizinische Universität Wien, Wien, Österreich

**Keywords:** Beziehung, Empathische Fürsorge, Autonomie, Aggression, Reflexion, Attachment, Empathic Care, Autonomy, Aggression, Reflection

## Abstract

Therapeutisches Wirken wird durch begünstigende Kontextfaktoren erleichtert, wobei therapeutische Interventionen gleichzeitig Anregung sein können, Kontextfaktoren zu verändern. Kommunikation und Therapie werden durch eine gute therapeutische Beziehung erst ermöglicht, vor allem unter erschwerten inneren und äußeren Bedingungen.

## Abstract (en)

Therapeutic action is facilitated by favourable contextual factors whereby therapeutic interventions can also be an incentive to change contextual factors. Communication and therapy are only made possible by a good patient–therapist relationship, especially under difficult internal and external conditions.

## Einleitung

Die therapeutische Beziehung ist als Wirkfaktor für den Therapieerfolg in den Fokus der Aufmerksamkeit gerückt. Wie aber wirkt sich der Kontext einer Behandlung auf die Gestaltung dieser Beziehung aus?

## Gesellschaftliche Perspektiven

Der gesellschaftliche Rahmen menschlichen Agierens ist ein kontinuierlicher Prozess, der von individuellen Handlungen getragen eigene Regeln und Regelmäßigkeiten zeitigt, die wiederum auf das Handeln der Akteure einwirken. Ein beschleunigter gesellschaftlicher Wandel beinhaltet Begriffe wie Globalisierung, Produktivitätssteigerung, Technisierung/Digitalisierung [1].

An Demokratisierungsprozesse knüpften politische Kämpfe an, um Freiheitsrechte einzufordern, aus welchen letztlich beispielsweise die PatientInnen-Empowerment-Bewegung hervorgegangen ist [2]. Paternalistische Sichtweisen innerhalb der Medizin wurden kritisiert und Zuschreibungen dem/r PatientIn als hilfsbedürftigem/r AkteurIn zurückgewiesen. Forderungen nach mehr Autonomie und einem Arzt-Patient-Verhältnis auf Augenhöhe wurden artikuliert [3]. Die Dominanz der ExpertInnen wird infrage gestellt, die Kompetenzen auf Seite der PatientInnen werden betont und wissenschaftliche Belege für diagnostisches und therapeutisches Tun gefordert [4].

Insgesamt wird eine Verschiebung vom Autoritäts- und Herrschaftsverhältnis zum Vertragsverhältnis konstatiert. Der Anspruch verändert sich: Aus dem Arzt-Patient-Verhältnis wird das Arzt-Kunden-Verhältnis [5]. Diese Perspektive birgt jedoch die Gefahr, die „Rationierung von Gesundheitsleistungen als ‚Marktgeschehen‘“ zu interpretieren [6]. Krankenbehandlung bleibt – trotz Kritik am Paternalismus – auf Wissensbestände und institutionalisierte Kompetenzen der BehandlerInnen angewiesen [7].

Gleichzeitig bilden PatientInnen keine passiven „Objekte“, sondern aktive Elemente ihrer eigenen Krankenbehandlung, treten als mehr oder weniger selbstbewusste Ko-ProduzentInnen [8] von Gesundheit auf; als „active patients“ [9], „informed patients“ [10] oder „patients as experts“ [11], deren Mitarbeit auch zunehmend eingefordert wird.

Die gelungene partnerschaftliche Beteiligung der PatientInnen ist voraussetzungsvoll

Insgesamt kann ein Wandel hin zu partizipativen Entscheidungsfindungen beobachtet werden, ohne dass paternalistische Elemente verschwinden. Vielmehr besteht beides fort als Pole eines Kontinuums. Sie werden je nach Behandlungssituation, Ausstattung der beteiligten Akteure, Art und Schweregrad der Erkrankung mehr oder weniger relevant und situativ aktiviert. In Kürze bleibt die Einsicht: Es gibt nicht „das“ Arzt-Patient-Verhältnis, sondern eine Vielzahl von Verhältnissen und Begegnungen im zunehmend fragmentierten Behandlungsnetzwerk [12]. „Ein einfaches Rezept für eine ‚gute Beziehung‘ ist daher kaum möglich“ [13]. Die gelungene partnerschaftliche Beteiligung der PatientInnen ist voraussetzungsvoll und fordert ein hohes Maß an affekt-kognitiver und körperlicher Leistungsfähigkeit *trotz* Erkrankung, aber z. B. auch ausreichende „Health Literacy“ [14, 15].

Trialogprozesse, die Angehörigenbewegungen inkludieren, sind noch nicht ausreichend als Elemente der Alltagskultur etabliert und Probleme sind hinsichtlich der Einführung eines entmetapsychologisierten Normierungssystems mit autonomen Technisierungsfolgen ohne systematische, prozesshafte Expertenprüfung angesprochen.

Zum Mangel an validen, nur im Sinnzusammenhang interpretierbaren Daten tritt die Herausforderung, den Informations-Overload [16] zu be- und verarbeiten. Aus verfügbaren Informationen belastbares Wissen zu integrieren, erfordert eine gelungene Health-Literacy-Strategie, die wiederum auf Basis einer guten therapeutischen Beziehung – Adhärenz – entstehen kann. Die Generierung diffuser Ängste durch kurze Fehlschlüsse oder Fake News und die paranoiden Exzesse aufgrund fehlender Expertenbewertung führen zu kaum zu kontrollierenden, hoch emotionalisierten Fanatisierungsprozessen, die faktenbasierte reflexive Mentalisierungsprozesse nötig machen durch vertrauenswürdige ExpertInnen in den globalen Informationscommunities.

Zweifellos hatte die COVID-19-Pandemie einen ungünstigen Einfluss auf die psychische Gesundheit [17–19], während Therapien durch Kontaktbeschränkungen erschwert waren. Maßnahmen zur Eindämmung der Pandemie (Ausgangsbeschränkungen, Quarantäne, Kontaktverfolgung) führten zu zahlreichen Einschränkungen in der individuellen Selbstbestimmung, zum Schutz von Leben unter dem Vorzeichen der Solidarität, zur Sicherung der Freiheit ohne Gefährdung der Gemeinschaft durch den Einzelnen [20, 21]. Krisenbedingt wurde das Online-Setting zur neuen Normalität. Die bisherige Studienlage zur Güte der therapeutischen Beziehung unter diesen Bedingungen zeigte, dass die Arbeitsallianz im Videotelefonie-Setting dem Face-to-face-Setting unterlegen war [22]. Longitudinale Studien versprechen allerdings einen besseren Einblick in die Folgen der Pandemie [19] und Folgen von Veränderungen des therapeutischen Settings als die bislang überwiegend durchgeführten Querschnittstudien, welche sich vor allem auf Selbstberichte (von betroffenen PatientInnen/TherapeutInnen) beschränken. Eine notwendige Adaptation therapeutischer Techniken und gleichzeitig das Vermeiden von Grenzverletzungen im Online-Setting können eine Herausforderung darstellen [23]. Lockdowns waren in Selbstberichten mit höherer Aggressionsbereitschaft, Ärger und Feindseligkeit in der Bevölkerung assoziiert, welche sich physisch und verbal äußerten [24]. Diese Entwicklung passt zur „Frustration-Aggression“-Hypothese von Berkowitz [25]: Wird die Erreichung eines gewünschten Zieles (Autonomie, Selbst-Effektivität, Beziehung/Bindung etc.) vereitelt, ist dies ausreichend für das Entstehen negativer Affektivität, welche dann eine Aggressionsneigung bedingt [24, 26]. Selbstberichtete Aggressivität muss sich nicht in entsprechendem Verhalten äußern, allerdings war während der Lockdowns auch Gewalt [27–30] dokumentiert worden. Die Empfehlung an TherapeutInnen, PatientInnen hinsichtlich Zeichen erhöhter Aggressivität zu evaluieren, gilt besonders während Lockdowns [24].

### Fallvignette

*„Ich habe diese Ordination gekauft. Die Ordination gehört mir, Sie gehören mir.“* Frau T ist eine unscheinbare Erscheinung. Auf dem Sessel in sich versunken und in schmuddelige Kleidung gehüllt, flüstert sie diese Worte wie zu sich selbst, zupft eine Haarsträhne vor das schmale Gesicht – wie einen Vorhang, zum Dahinterverschwinden. Frau T, 25-jährig und seit 2 Jahren arbeitslos, hat ihre Lehre zur Verkäuferin abgebrochen; sie lebt seither sozial zurückgezogen aufgrund von paranoiden Ängsten. Als der Behandler nach dem Befinden von Frau T fragt, fängt diese plötzlich an zu schreien, wobei sie perseveriert, diese Ordination gekauft zu haben und insgesamt für eine große Aufgabe berufen zu sein. Ohne ersichtlichen Grund scheint sie sich kurz danach wieder beruhigt zu haben und gibt nun Anweisungen an den Behandler, einen Beschwerdebrief zu schreiben, da man versuche, sie zu vergiften. Als der Behandler dieser Anordnung nicht unmittelbar nachkommt, betastet Frau T das eigene Gesicht. *„Ich verliere mein Gesicht. Mein Gesicht ist weg.“ *Frau T rutscht nervös auf dem Sessel hin und her und scheint zu überlegen, die Stirn ist in tiefe Falten gerunzelt, die Augen dem Blick des Behandlers stets ausweichend. Weiterhin sei sie davon überzeugt, verfolgt und vergiftet worden zu sein, *„gesichtsauflösende Medikamente“* seien die Ursache, sie selbst habe sie sich verschrieben, beschließt sie den Dialog.

### Diskussion

Zu Beginn der Intervention steht die Etablierung einer therapeutischen Beziehung, welche empathisches sich Einfühlen und Mentalisieren erfordert. Wie herausfordernd dies sein kann, wird besonders in dieser Begegnung mit einer Patientin sichtbar, welche externalisierend, projektiv-identifizierend und mittels Allmachtfantasien ihre Affekte reguliert. Affektives Containment und strukturierende Interventionen, welche Sicherheit und (Selbst‑)Kontrolle vermitteln, sind nun notwendig [31, 32]. Den dominanten Affekt zu adressieren, stellt eine weitere effektive Intervention dar [33]. Der analytikerzentrierten [34] Intervention [35] im Hier und Jetzt (der therapeutischen Beziehung) kommt eine entscheidende Rolle zu. Die Begegnung von Frau T mit dem Behandler fand nicht im Videotelefonie-Setting statt.

Strukturierende Interventionen, die Sicherheit und (Selbst‑)Kontrolle vermitteln, sind notwendig

Unabhängig vom Kontext, sind Behandler allerdings gefordert, die negativen Affekte im Jetzt der therapeutischen Beziehung zu „containen“, um die weitere therapeutische Arbeit zu ermöglichen. Erst auf Basis einer stabilen therapeutischen Beziehung ist das notwendige Durcharbeiten negativer Spitzenaffekte möglich [36].

Eine besondere Herausforderung stellt der Umgang mit Aggressivität dar, sei sie passiv/unterschwellig oder offen geäußert. Im Fall der Eskalation von Gewalt, der Selbst- oder Fremdgefährdung kommt der/die FachärztIn für Psychiatrie in die Situation, entsprechend den Kriterien des Unterbringungsgesetzes (UBG) paternalistisch für Patienten zu entscheiden und ihre Freiheit zu ihrem eigenen Wohl zu beschneiden. Als Gründe für Aggression im Gesundheitswesen zeigen sich auf Patientenseite ein hohes inneres Spannungsniveau und Aggressionspotenzial, mangelnde Einsicht und die Wahrnehmung von Maßnahmen als Gewalt anstatt Behandlung, eine eingeschränkte Kommunikationsmöglichkeit bei gleichzeitig hohem Erwartungsdruck [37]. Wird ein Verlust an Autonomie, Kontrolle, Ungerechtigkeit oder Hilflosigkeit erlebt, nimmt das Aggressionspotenzial zu. Erlittene Kränkungen und Demütigungen, Bestrafungen, die Verarbeitung von Trauer/Verlust oder Minderwertigkeitsgefühlen tragen zu Ärger, Gewalt und Aggression bei. BehandlerInnen sind aufgefordert, erste Anzeichen von Gewalt wahrzunehmen, Ruhe zu bewahren und die eigene Sicherheit zu beachten, wobei Körpersprache beobachtet und Blickkontakt hergestellt werden sollte [38]. Weder selbst zu provozieren noch sich provozieren zu lassen und eine wertschätzende Haltung zu bewahren, um Bedürfnisse und Gefühle des Gegenübers wahrnehmen und herausarbeiten zu können, bedarf einer ausreichenden Kommunikations- und Deeskalationskompetenz.

Kommunikationskompetenz, Mentalisierungsfähigkeit und Arbeitsumfeld wirken zusammen

Aggressive Kommunikation kann als Beziehungsversuch des/r PatientIn wahrgenommen werden, welcher in Ermangelung anderer Umgangsweisen mit Ärger und Wut in Erscheinung tritt. Neben Kommunikationskompetenz und Mentalisierungsfähigkeit trägt das Arbeitsumfeld dazu bei, ob Menschen in Gesundheitsberufen ihre volle Kompetenz in der Interaktion mit aggressiven PatientInnen entfalten können. Dauerfrustration, chronischer Stress, zahlreiche und hohe Spitzenstressoren tragen dazu bei, dass Empathie, Toleranz, Differenzierungsfähigkeit und soziale Kompetenzen schwinden können [37]. Neben dem Schaffen entsprechender Arbeitsbedingungen wirkt sich eine Stärkung der Mentalisierungsfähigkeit in stressreichen, emotionalisierten Situationen positiv aus.

Psychotherapeutische Interventionen zur Verbesserung von Schwierigkeiten im sozialen Umgang fokussieren zunehmend auf eine Verbesserung der Emotionsregulation und der kognitiven Fähigkeiten, welche für Anhedonie und Apathie relevant sind (e.g. Positive Emotions Program for Schizophrenia (PEPS) [39]). Die Güte der Emotionswahrnehmung, die kognitive Leistung einschließlich der sozialen Fähigkeiten sowie die klinische Symptomatik sind signifikante Prädiktoren der Mentalisierungsfähigkeit schizophrener Patienten [40]. Kontextfaktoren sind für die therapeutische Allianz relevant [41].

## Fazit für die Praxis

- Die Beziehung zwischen ÄrztIn und PatientIn findet im Spannungsfeld zwischen Ethik der Fürsorge und Ethik der Autonomie statt.
- Professionelles Deeskalationsmanagement ist im Umgang mit Gewalt und Aggression frühzeitig und zielgerichtet indiziert.
- Ohne Basis einer guten Bindung ist Kommunikation erschwert. Vor allem indizierte psychotherapeutische Interventionen lassen sich ohne etablierte Beziehung nicht adäquat einsetzen.
- Das Setting kann Therapieerfolge fördern oder erschweren.

## References

[1] Hobsbawm E (1998). Das Zeitalter der Extreme. Weltgeschichte des 20. Jahrhunderts.

[2] Bröckling U (2019). Das unternehmerische Selbst. Soziologie einer Subjektivierungsform.

[3] Coulter A (2002). The autonomous patient. Ending paternalism in medical care.

[4] Eibach U (1997). Vom Paternalismus zur Autonomie des Patienten? Medizinische Ethik im Spannungsfeld zwischen einer Ethik der Fürsorge und einer Ethik der Autonomie. Z Med Ethik.

[5] Ewert B (2013). Vom Patienten zum Konsumenten? Nutzerbeteiligung und Nutzeridentitäten im Gesundheitswesen.

[6] Kloiber O (2000). Patienten sind keine Kunden. Dtsch Arztebl.

[7] Freidson E (1979). Der Ärztestand. Berufs- und wissenschaftssoziologische Durchleuchtung einer Profession.

[8] Pelikan JM, Griessler E, Grundböck A, Krajic K, Pelikan JM, Stacher A, Grundböck A, Krajic K (1998). Das virtuelle Krankenhaus – Ausweg oder Königsweg für die Krankenversorgung der Zukunft?. Virtuelles Krankenhaus zu Hause – Entwicklung und Qualität von Ganzheitlicher Hauskrankenpflege: Theoretische Konzepte, gesundheitspolitischer Kontext und praktische Erfahrungen in Europa.

[9] Heldal F, Tjora A (2009). Making sense of patient expertise. Soc Theory Health.

[10] Kivits J (2004). Researching the ‘Informed Patient’. The case of online health information seekers. Inf Commun Soc.

[11] Prior L (2003). Belief, knowledge and expertise: the emergence of the lay expert in medical sociology. Sociol Health Illn.

[12] Vogd W (2006). Von der Organisation Krankenhaus zum Behandlungsnetzwerk? Untersuchungen zum Einfluss von Medizincontrolling am Beispiel einer internistischen Abteilung. Berl J Soziol.

[13] Begenau J, Schubert C, Vogd W (2010). Einleitung: Die Arzt-Patient-Beziehung aus soziologischer Sicht. Die Arzt-Patient-Beziehung.

[14] Lenartz N, Soellner R, Rudinger G, Jungbauer M (2016). Health Literacy. Entwicklung und Bedeutung einer Schlüsselkompetenz für gesundheitsgerechtes Leben. Handbuch Gesundheitssoziologie.

[15] Siegrist J (2005). Medizinische Soziologie.

[16] Esposito E (1993). Der Computer als Medium und Maschine. Z Soziol.

[17] Wu T, Jia X, Shi H, Niu J, Yin X, Xie J, Wang X (2021). Prevalence of mental health problems during the COVID-19 pandemic: a systematic review and meta-analysis. J Affect Disord.

[18] Salari N, Khazaie H, Hosseinian-Far A, Khaledi-Paveh B, Kazeminia M, Mohammadi M, Shohaimi S, Daneshkhah A, Eskandari S (2020). The prevalence of stress, anxiety and depression within front-line healthcare workers caring for COVID-19 patients: a systematic review and meta-regression. Hum Resour Health.

[19] The Lancet Psychiatry (2021). COVID-19 and mental health. Lancet Psychiatry.

[20] Mitscherlich Schönherr O (2021) Freiheit in Krisenzeiten. https://www.br.de/radio/bayern2/sendungen/zum-sonntag/zum-sonntag-mitscherlich-schoenherr-100.html (BR Bayern 2). Zugegriffen: 23.12.2021

[21] DGGG (2020) Statement der DGGG. Pressemitteilung vom 24.4.2020. https://www.sozial.de/schutz-ja-paternalismus-nein!.html. Zugegriffen: 23.12.2021

[22] Simpson SG, Reid CC (2014). Therapeutic alliance in videoconferencing psychotherapy: a review. Aust J Rural Health.

[23] Eichenberg C (2020). Psychotherapie in der Coronakrise. Trendwende in der Online-Psychotherapie. Dtsch Arzteblatt PP.

[24] Killgore WDS, Cloonan SA, Taylor EC, Anlap I, Dailey NS (2021). Increasing aggression during the COVID-19 lockdowns. J Affect Disord Rep.

[25] Berkowitz L (1989). Frustration-aggression hypothesis: examination and reformulation. Psychol Bull.

[26] Breuer J, Elson M, Sturmey P (2017). Frustration-aggression theory. The Wiley handbook of violenceand aggression.

[27] Eichenberg C (2021). Onlinepsychotherapie in Zeiten der Coronapandemie. Psychotherapeut.

[28] Leslie E, Wilson R (2020). Sheltering in place and domestic violence: evidence from calls for service during COVID-19. J Public Econ.

[29] Lawson M, Piel MH, Simon M (2020). Child maltreatment during the COVID-19 pandemic: consequences of parental job loss on psychological and physical abuse towards children. Child Abuse Negl.

[30] Sherman WF, Khadra HS, Kale NN, Wu VJ, Gladden PB, Lee OC (2021). How did the number and type of injuries in patients presenting to a regional level I trauma center change during the COVID-19 pandemic with a stay-at-home order?. Clin Orthop Relat Res.

[31] Löffler-Stastka H, Blueml V, Boes C (2010). Exploration of personality factors and their predictive impact on therapy utilization: the externalizing mode of functioning. Psychother Res.

[32] Livesley WJ, Jang KL, Vernon PA (1998). Phenotypic and genetic structure of traits delineating personality disorder. Arch Gen Psychiatry.

[33] Clarkin JF, Yeomans F, Kernberg OF (2006). Psychotherapy for borderline personality.

[34] Steiner J, Schafer R (1997). Problems of psychoanalytic technique: patient centered and analyst-centered interpretations. The contemporary Kleinians of London.

[35] Clarkin JF, Levy KN, Lenzenweger MF, Kernberg OF (2007). Evaluating three treatments for borderline personality disorder: a multiwave study. Am J Psychiatry.

[36] Datz F, Wong G, Löffler-Stastka H (2019). Interpretation and working through contemptuous facial micro-expressions benefits the patient-therapist relationship. Int J Environ Res Public Health.

[37] Löffler-Stastka H (2021). Kommunikativer Umgang mit Gewalt im Gesundheitswesen.

[38] Wesuls R, Heinzmann T, Brinker L (2005). Professionelles Deeskalationsmanagement (ProDeMa). Praxisleitfaden zum Umgang mit Gewalt und Aggression in den Gesundheitsberufen.

[39] Favrod J, Nguyen A, Tronche A-M, Blanc O, Dubreucq J, Chereau-Boudet I, Capdevielle D, Llorca Pierre M (2019). Impact of positive emotion regulation training on negative symptoms and social functioning in schizophrenia: a field test. Front Psychiatry.

[40] Vaskinn A, Andersson S, Østefjells T, Andreassen OA, Sundet K (2018). Emotion perception, non-social cognition and symptoms as predictors of theory of mind in schizophrenia. Compr Psychiatry.

[41] Norwood C, Moghaddam NG, Malins S, Sabin-Farrell R (2018). Working alliance and outcome effectiveness in videoconferencing psychotherapy: a systematic review and noninferiority meta-analysis. Clin Psychol Psychother.

